# VDR Variants rather than Early Pregnancy Vitamin D Concentrations Are Associated with the Risk of Gestational Diabetes: The Ma'anshan Birth Cohort (MABC) Study

**DOI:** 10.1155/2019/8313901

**Published:** 2019-06-24

**Authors:** Beibei Zhu, Kun Huang, Shuangqin Yan, Jiahu Hao, Peng Zhu, Yao Chen, Aoxing Ye, Fangbiao Tao

**Affiliations:** ^1^Department of Maternal, Child & Adolescent Health, Anhui Medical University, Hefei, China; ^2^Anhui Provincial Key Laboratory of Population Health & Aristogenics, Anhui Medical University, Hefei, China; ^3^Ma'anshan Maternal and Child Health Care Center, Ma'anshan, China

## Abstract

**Aim:**

This study investigated the associations among early pregnancy vitamin D concentrations, seasonality, and vitamin D metabolic gene variants and how these variables related alone and in interaction with the risk of gestational diabetes mellitus (GDM).

**Methods:**

Research participants were women from the Ma'anshan birth cohort study in China. The overall study included 3110 women to explore the association between early pregnancy vitamin D concentrations and the risk of GDM. In the current analysis, a nested case-control study of 274 GDM cases and 380 controls was conducted to investigate seven vitamin D metabolic gene variants and the risk of GDM. Vitamin D concentrations were measured by radioimmunoassay. Genotypes were determined by improved multiple ligase detection reaction. Interactions between genetic variants and vitamin D as predictors of the risk of GDM were evaluated by a pair-wise analysis under a multiplicative interaction model.

**Results:**

Vitamin D concentrations were not significantly associated with the risk of GDM (OR = 0.79, 95% CI = 0.55-1.13) after adjusting for seasonality. Fall-winter conceptions had a 37% decreased risk of GDM compared with spring-summer conceptions (OR = 0.63, 95% CI = 0.49-0.81), independent of vitamin D concentrations. Two VDR gene variants rs1544410 (OR = 2.03, 95% CI = 1.17-3.51 for CT versus CC) and rs731236 (OR = 2.42, 95% CI = 1.29-4.55 for GA versus AA) were significantly associated with the risk of GDM. No interactions among genetic variants and vitamin D concentrations were detected.

**Conclusion:**

Early pregnancy vitamin D insufficiency or deficiency was not significantly associated with the risk of GDM. The results of this study emphasize the importance of genetic variants in VDR and conception season as factors that affect the risk of GDM.

## 1. Introduction

Gestational diabetes mellitus (GDM) is one of the most common complications during pregnancy and is therefore a significant public health issue. The global prevalence of GDM remains unknown, as no systematical synthesized data is available [1], but GDM is estimated to affect 15~20% of pregnancies [2]. GDM brings both short- and long-term threats to the health of mothers [3] and their children [4]. Nevertheless, the etiology of GDM is not fully understood, as only a few risk factors have been established, including advanced maternal age, obesity, and a family history of diabetes [5].

Vitamin D insufficiency and deficiency a global concern [6], especially for pregnant women [7], and the association between vitamin D levels and the GDM risk have received considerable research attention [8, 9]. The varied results from observational studies [10] and randomized trails [11] reveal that vitamin D concentrations are influenced by multiple factors and that the association between vitamin D levels and GDM is likely confounded by these factors. The most important factors are body mass index (BMI) and seasonality. BMI is a well-established risk factor for GDM [12] and being closely related to serum vitamin D concentrations [13]. Seasonality and vitamin D concentrations are also intuitively intertwined [13]. Some evidence suggests that seasonality is independently linked to glucose homeostasis in pregnancy [14]. The majority of existing observational studies have inadequately adjusted for these two factors, and only a few studies have simultaneously adjusted both BMI and seasonality, which has led to inconsistent results [8, 15]. Well-designed studies with a sample size that is large enough to evaluate these variables are desperately needed to determine whether pregnant women could benefit from vitamin D supplementation to reduce risk of GDM.

Genetic variants in vitamin D metabolic genes are good candidates to better understand how vitamin D pathways are involved in the pathogenesis of GDM. Several studies [16, 17] have contributed data to this subject. However, to our knowledge, no research to date has explored the interactions of variants in vitamin D metabolic genes with vitamin D concentration on the risk of GDM. Given these considerations, the current study used data from the Ma'anshan birth cohort (MABC) study in China to explore both the independent effects and the interactions of vitamin D in early pregnancy and seven genetic variants on vitamin D metabolic genes on the risk of GDM.

## 2. Material and Methods

### 2.1. Study Population

**Figure 1:**
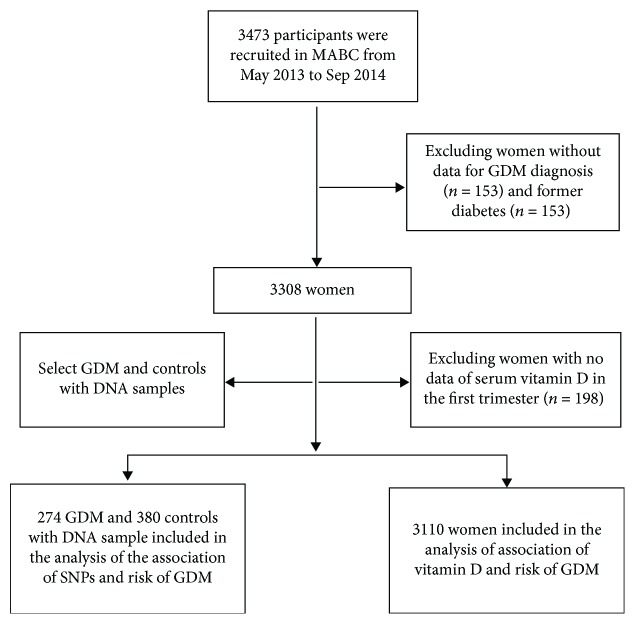
Flow chart of participant inclusion and exclusion of our study.

The Ma'anshan birth cohort (MABC) study is a population-based prospective study conducted in Ma'anshan city of Anhui province in China [18]. The MABC recruited 3 474 pregnant women at their first prenatal visit between May 2013 and September 2014. Participants were then followed until labor for a maximum of four times (the first, second, and third trimesters and delivery). Of these participants, 3 110 women who had both a diagnosis of GDM (or not) and vitamin D assessment during the first trimester were considered as candidates for the current study. To examine the specific associations between single nucleotide polymorphisms (SNPs) and the risk of GDM, 274 GDM cases and 380 age-matched controls were included (see Figure 1 for a flow chart). The present study was approved by the ethics committee of Anhui Medical University. Written informed consent was obtained from all pregnant women.

### 2.2. Data Collection

Extensive data were collected using a structured self-report questionnaire that was administered by trained interviewers at every visit. These data included age, race, education, socioeconomic status, smoking, alcohol consumption, and anthropometric measures. Information on multivitamin supplements was assessed for different periods (one month before pregnancy, first/second/third trimester of pregnancy). If supplement used was indicated, frequency (e.g., daily, weekly) was assessed.

### 2.3. GDM Diagnosis

At 24~28 weeks of gestation, women were screened by a “one-step” standardized 75 g oral glucose tolerance test (OGTT) to diagnose GDM. Venous blood samples were collected at 0, 1, and 2 h after a glucose load, and a positive diagnosis of GDM was made when any of the following criteria were met: fasting plasma glucose at 0 h ≥5.1 mmol/l, at 1 h ≥10 mmol/l, and at 2 h ≥8.5 mmol/l [19].

### 2.4. Vitamin D Measurement

Blood sample was collected before 14 weeks of the first trimester. Fasting blood was drawn in the morning before 10 am and stored at -80 degrees. Serum 25-hydroxyvitamin D (25[OH] D) concentration, a vitamin D metabolite, was measured by radioimmunoassay (RIA) using a commercially available kit (DiaSorin, Stillwater, Minnesota 55082-0285, USA). Samples were randomly assayed and measured in a blinded manner. For quality control, standard substances of low (10.9~23.3 ng/ml) and high (36.1~76.5 ng/ml) concentrations were both determined using our method, which was proven to be reliable. The intra-assay and interassay coefficients of variation of 25[OH] D were both <10%. All participants had 25[OH] D concentration above the detection limit (1.5 ng/ml).

### 2.5. SNP Selection and Genotyping

By tracking the literature, we selected seven SNPs that are commonly reported in the vitamin D metabolism pathway for assessment. These SNPs included rs1544410 and rs731236 in the vitamin D receptor (VDR), rs2282679 and rs7041 in the vitamin D-binding protein (DBP), rs3829251 in 7-dehydrocholesterol reductase (DHCR7), rs6013897 in the cytochrome P450 family 24 subfamily A member 1 (CYP24A1), and rs6599638 in chromosome 10 open reading frame 88 (C10orf88). Genotyping of candidate SNPs was conducted by the improved multiple ligase detection reaction (iMLDR), with technical support from the Center for Human Genetics Research, Shanghai Genesky Biotechnology Company. For quality control, 5% of duplicate samples were independently reanalyzed in a blinded manner. SNPs that violated the Hardy-Weinberg equilibrium (HWE) in controls would be removed from the analysis. The call rate of these SNPs was required to be over 90%.

### 2.6. Statistical Analysis

Prepregnancy body mass index (BMI) was categorized into four groups: <18.5, 18.5~24, 24~28, and ≥28 [20]. Vitamin D concentrations were classified into three categories: deficiency, 25[OH] D <20 ng/ml; insufficiency, 25[OH] D 20~30 ng/ml; and normal/optimal, 25[OH] D >30 ng/ml. Smoking at early pregnancy was defined as either ongoing smoking or former smoker who quit after knowing about pregnancy. Alcohol drinking was categorized as never, occasional, and regular. Multivitamin supplement intake frequency was categorized as never, 1~2 times/week, 3~6 times/week, and every day. Education level was categorized into five groups: primary school or below, middle school, high school, junior college, and undergraduate or above. Monthly income levels (Chinese yuan) were divided into four groups: <1000, 1000~2500, 2500~4000, and ≥4000. The Northern Hemisphere seasons were defined as spring (February to April), summer (May to July), fall (August to October), and winter (November to January).

To examine the group differences, the chi-square test was used for categorical variables, whereas either Student's *t*-test or ANOVA was used for continuous variables. Effect estimates for vitamin D deficiency, conception season, and genetic variants alone and in combination, as risk factors for GDM, were evaluated using multivariate logistic regression. The most complete model included potential confounders such as age, prepregnancy BMI, family diabetes history, parity and smoking at early pregnancy, drinking status, gestational week, education, and income. Stratified analyses of vitamin D concentrations were conducted according to different genotypes. The probability value for interactions between SNPs and vitamin D was calculated by a pair-wise analysis under the multiplicative interaction model which was evaluated by a likelihood ratio test in the logistic regression.

Analyses were conducted using SPSS v16.0, and statistical significance was set at *P* < 0.05.

## 3. Results

Among the full sample of 3 110 women, we documented 399 cases of GDM.  Table 1 shows the characteristics of total participants and different characteristics according to GDM status. GDM cases were more likely to have higher prepregnancy BMI, advanced age, family history of type 2 diabetes, a conception season of summer, and higher vitamin D concentrations.

The highest vitamin D concentrations were seen in pregnancies with summer conception; the lowest occurred in winter. Multivitamin intake in early pregnancy was associated with increased vitamin D concentration in the first trimester. The factors of gestational weeks, parity, prepregnancy BMI, education, and smoking status in early pregnancy also influenced its concentrations (see Supplementary Tables 1 and 2).

Table 2 shows the effect sizes of vitamin D concentration in the first trimester and conception season on the risk of GDM, based on logistic regression analysis. Vitamin D deficiency was associated with a decreased risk of GDM relative to normal vitamin D concentrations. However, when the model was adjusted for conception season (OR = 0.79, 95% CI = 0.55-1.13) or season of the blood draw (OR = 0.78, 95% CI = 0.54-1.11), the association became nonsignificant. In the most adjusted model, conception in fall-winter had a 37% decreased risk of GDM (OR = 0.63, 95% CI = 0.49-0.81), independent of vitamin D concentrations, compared with spring-summer (see Supplementary Table 3).

Table 3 indicates that both SNPs rs1544410 and rs731236 in VDR were significantly associated with the risk of GDM. Compared with rs1544410-CC genotypes, individuals who carried CT genotypes had an increased risk of GDM (OR = 2.03, 95% CI = 1.17-3.51). Compared with rs731236-AA genotypes, individuals who carried GA genotypes had an increased risk of GDM (OR = 2.42, 95% CI = 1.29-4.55). Supplementary Table 3 presents the stratified analysis of the association of vitamin D concentrations with the risk of GDM according to different SNP genotypes. No significant interactions were detected between SNPs and vitamin D concentrations, with respect to the risk of GDM.

## 4. Discussion

The current large prospective cohort study indicated that vitamin D concentrations in early pregnancy are not independently associated with the risk of GDM but are confounded by seasonality. The conception season of fall-winter was significantly associated with a decreased risk of GDM independent of vitamin D concentration. Furthermore, the current study demonstrated significant associations of the two SNPs rs1544410 and rs731236 in the VDR gene with the risk of GDM.

The large sample size of our study enabled us a comprehensive evaluation of vitamin D status among pregnant women. Consistent with other research [7–9], a high rate of vitamin D deficiency and insufficiency was observed. Vitamin D concentrations also demonstrated expected relationships with season and multivitamin supplement intake. In contrast with other research which supports an association between higher BMI and lower vitamin D [21], this study found a relatively small positive association between vitamin D concentration and prepregnancy BMI. This result was consistent with the studies conducted in China [22] and in the Czech Republic [23]. Moreover, smoking in the early pregnancy exerted a negative effect on vitamin D concentration, and its effect size was the largest among all studied factors.

We did not find that vitamin D concentrations in early pregnancy were significantly associated with the risk of GDM, but conception season was. Vitamin D concentrations and the risk of GDM have been controversial, in part as the results from observational studies and randomized controlled trials have been inconsistent. Vitamin D concentrations and the risk of GDM are influenced by multiple shared factors, so controlling confounding variables might be the priority to guarantee reliability. Unfortunately, previous studies have typically had obvious confounding factors such as seasonality. The current study was large enough and had sufficient measurement of constructs to suggest that higher vitamin D concentrations had a simple association with an increased risk of GDM; the association vanished after adding conception season to the model. This result is supported by many studies [24–27], especially those that have controlled for seasonality [26, 27]. While the current results are supported by large sample size and many aspects of measurement, the study did adopt a common definition for vitamin D insufficiency and deficiency. There are other definitions, however, such as the more conservative definition adopted by the Institute of Medicine, which uses the following categorization system: <12 ng/ml is “risk of deficiency,” 12~20 ng/ml is “risk of inadequacy,” and >20 ng/ml is “sufficiency” [28]. The interpretation of the current study should bear in mind alternative models of classification.

The seasonality and risk/prevalence of GDM have become an emerging research interest lately [29–32]. This is the first study that revealed that the association between vitamin D concentrations and the risk of GDM might partly be confounded by seasonality and in particular that summer conception (in the Northern Hemisphere) increases the risk of GDM independent of vitamin D concentration and other potential confounders. The mechanism behind this association remains unclear. One potential mechanism is that there may be other third variables or modifiers related to the season, such as physical activity [33] or air pollution [34], however, in our study that might not be the case. A second possibility relates to circannual rhythms. For example, the duration of the peak of melatonin secretion is positively correlated with the length of the night period [35], and there is evidence that lowering the melatonin is associated with higher risk of diabetes [36]. Summer nights are much shorter than in winter, so summer conception could increase the risk of GDM. However, our result was opposite to some other studies [30, 31] which showed that winter conception was a risk factor to GDM. The current results are consistent with a study conducted in the UK which found that type 1 diabetes was more frequently diagnosed in winter [37]. These conflicting results suggest a complicated mechanism between seasonality and GDM and warrant further exploration.

Genetic background inspection could provide novel information about the vitamin D metabolic pathway in the pathogenesis of GDM. Two SNPs in the VDR gene were found in the current study to be correlated with an increased risk of GDM. VDR gene encodes vitamin D3 receptor, which is a member of the nuclear hormone receptor superfamily and mediates the actions of vitamin D [38]. SNPs on this gene have been extensively studied [39] and are widely associated with a variety of metabolic phenotypes [40–42]. Among these SNPs, rs1544410 and rs731236 were commonly reported as significant, especially as they influence insulin resistance-related diseases [43]. Rs1544410 is an intron variant, and rs731236 is a synonymous variant, while the minor allele frequency (MAF) of both two SNPs varies across different races and ethnic groups, with lowest among East Asian to highest among European, which could partly explain the inconsistency across the diverse population. In our study, for rs1544410 and rs731236, different genotypes have marginal differences in vitamin D concentrations but they are significantly associated with the risk of GDM. However, for the two other SNPs of rs2282679 and rs6599638, the pattern is reversed. Based on these data, it was speculated that the relationships among the VDR variants and vitamin D concentrations would not necessarily explain the associations between the VDR variants and the risk of GDM. The association of the VDR variants with vitamin D concentration can instead be a marker of the (unclear) effects of the variants that modify the risk of GDM. The current lack of significant interactions between vitamin D concentrations and genetic variants supports the idea that genetic variants might be more important than vitamin D concentrations when considering vitamin D metabolic pathways involved in GDM, and our results are entirely supported by another study on a similar topic about type 1 diabetes [44].

The current study has several notable strengths. It had a large sample size which permitted the consideration of several critical and simultaneous potential confounders. Second, the study was able to evaluate both the independent role of vitamin D concentrations and interactions with SNPs in vitamin D metabolic gene as risk factor for GDM. Despite these advantages, limitations of the study include lack of information on diet, physical activity, and the assessment of a limited number of SNPs. Also, the vast majority of the current sample was Han Chinese, which limits the study's generalizability.

In conclusion, this study did not find that early pregnancy vitamin D insufficiency or deficiency was significantly associated with the risk of GDM. Instead, the current results emphasize the importance of genetic variants in VDR and conception season as risk factors for GDM. Although the current study contributes to the understanding of vitamin D metabolic pathway in the occurrence of GDM, other well-designed studies are needed to replicate and extend these results.

## Figures and Tables

**Table 1:** Characteristics of women included for investigating the association of vitamin D concentrations and the risk of GDM.

Variables	Total	Non-GDM (*N* = 2711)	GDM (*N* = 399)	*P*
*Age*	26.7 ± 3.7	26.5 ± 3.5	28.1 ± 4.4	**<0.001**
				**<0.001**
<25	910 (29.3)	832 (30.7)	78 (19.5)	
25~30	1634 (52.5)	1438 (53.0)	196 (49.1)	
30~35	434 (14.0)	351 (12.9)	83 (20.8)	
≥35	132 (4.2)	90 (3.3)	42 (10.5)	
*Prepregnancy BMI*	20.9 ± 2.8	20.7 ± 2.7	22.3 ± 3.2	**<0.001**
				**<0.001**
<18.5	577 (18.6)	545 (20.1)	32 (8.0)	
18.5~24	2154 (69.3)	1882 (69.4)	272 (68.2)	
24~28	306 (9.8)	234 (8.6)	72 (18.0)	
≥28	72 (2.3)	49 (1.8)	23 (5.8)	
*Gender of infant*				0.797
Male	1563 (50.3)	1367 (50.4)	196 (49.1)	
Female	1502 (48.3)	1309 (48.3)	193 (48.4)	
Unknown	45 (1.4)	35 (1.3)	10 (2.5)	
*Parity*				**0.003**
Nulliparous	2751 (88.5)	2416 (89.1)	335 (84.0)	
Multiparous	359 (11.5)	295 (10.9)	64 (16.0)	
*Education*				0.554
Primary school or below	39 (1.3)	33 (1.2)	6 (1.5)	
Middle school	595 (19.1)	507 (18.7)	88 (22.1)	
High school	699 (22.5)	611 (22.5)	88 (22.1)	
Junior college	964 (31.0)	844 (31.1)	120 (30.1)	
Undergraduate or above	813 (26.1)	716 (26.4)	97 (24.3)	
*Monthly income (Chinese yuan)*				0.087
<1000	53 (1.7)	45 (1.7)	8 (2.0)	
1000~2500	771 (24.8)	655 (24.2)	116 (29.1)	
2500~4000	1338 (43.0)	1167 (43.0)	171 (42.9)	
>4000	948 (30.5)	844 (31.1)	104 (26.1)	
*Smoking in early pregnancy*				0.670
No	2982 (95.9)	2601 (95.9)	381 (95.5)	
Yes	128 (4.1)	110 (4.1)	18 (4.5)	
*Drinking*				0.764
Never	2865 (92.1)	2495 (92.0)	370 (92.7)	
Occasionally	240 (7.7)	212 (7.8)	28 (7.0)	
Regularly	5 (0.2)	4 (0.1)	1 (0.3)	
*Family history of diabetes*				**<0.001**
No	2439 (78.4)	2155 (79.5)	284 (71.2)	
Yes	255 (8.2)	198 (7.3)	57 (14.3)	
Uncertain	416 (13.4)	358 (13.2)	58 (14.5)	
*Gestational weeks in the 1^st^ trimester*	10.0 ± 2.1	10.1 ± 2.2	10.0 ± 2.0	0.660
*Season of conception*				**<0.001**
Spring	929 (29.9)	793 (29.3)	136 (34.1)	
Summer	916 (29.5)	773 (28.5)	143 (35.8)	
Fall	643 (20.7)	581 (21.4)	62 (15.5)	
Winter	622 (20.0)	564 (20.8)	58 (14.5)	
*Season in the 1^st^ trimester*				**< 0.001**
Spring	713 (22.9)	630 (23.2)	83 (20.8)	
Summer	1187 (38.2)	998 (36.8)	189 (47.4)	
Autumn	549 (17.7)	482 (17.8)	67 (16.8)	
Winter	661 (21.3)	601 (22.2)	60 (15.0)	
*Serum 25[OH] D*	18.2 ± 8.4	18.0 ± 8.4	19.8 ± 8.3	**<0.001**
				**0.002**
Deficiency	2052 (66.0)	1819 (67.1)	233 (58.4)	
Insufficiency	747 (24.0)	635 (23.4)	112 (28.1)	
Normal	311 (10.0)	257 (9.5)	54 (13.5)	
*Fasting glucose*	4.3 ± 0.6	4.2 ± 0.4	5.0 ± 0.7	**<0.001**
*1 h glucose load*	7.2 ± 1.8	6.8 ± 1.4	9.6 ± 2.0	**<0.001**
*2 h glucose load*	6.1 ± 1.3	5.9 ± 1.0	7.7 ± 1.9	**<0.001**

Abbreviation: BMI, body mass index.

**Table 2:** The association between vitamin D concentration in the first trimester, conception season, and risk of GDM.

Variable	Crude OR (95% CI)	Adjusted OR1 (95% CI)	Adjusted OR2 (95% CI)	Adjusted OR3 (95% CI)
*Vitamin D*				
Normal	1.00	1.00	1.00	1.00
Insufficiency	0.84 (0.59-1.20)	0.86 (0.59-1.26)	0.88 (0.60-1.29)	0.87 (0.55-1.27)
Deficiency	**0.61 (0.44-0.84)**	**0.69 (0.49-0.98)**	0.79 (0.55-1.13)	0.78 (0.54-1.11)
*Conception season*				
Spring-summer	1.00	1.00	1.00	—
Fall-winter	**0.59 (0.47-0.74)**	**0.61 (0.48-0.77)**	**0.63 (0.49-0.81)**	—

Adjusted OR1 for vitamin D is adjusted for age, prepregnancy BMI, family history of diabetes, parity, smoking, drinking, gestational week of blood withdrawn, income, and education; adjusted OR1 for conception season is adjusted for age, prepregnancy BMI, family history of diabetes, parity, smoking, drinking, income, and education; adjusted OR2 for vitamin D is adjusted OR1 plus adjusting for conception season (as four categories); adjusted OR2 for conception season was adjusted OR1 plus adjusting for vitamin D (as three categories). Adjusted OR3 is adjusted OR1 plus the season of the blood draw (as four categories) in the first trimester. Abbreviations: BMI: body mass index; OR: odds ratio; CI: confidence interval.

**Table 3:** The association between SNPs in vitamin D metabolism genes and the risk of GDM.

SNPs	Gene	Controls/cases	*P* ^∗^	Vitamin D	*P* ^†^	Crude OR (95% CI)	Adjusted OR (95% CI)
*rs1544410*	VDR		**0.021**		0.067		
CC		353/240		19.35 ± 8.72		1.00	1.00
CT		27/34		17.09 ± 8.52		1.85 (1.09-3.15)	2.03 (1.17-3.51)
*rs731236*	VDR		**0.006**		0.059		
AA		341/237		19.45 ± 8.78		1.00	1.00
GA		18/29		16.72 ± 7.47		2.32 (1.26-4.28)	2.42 (1.29-4.55)
*rs7041*	GC		0.226		0.063		
AA		170/107		18.16 ± 8.24		1.00	1.00
CA		158/133		19.70 ± 8.64		1.34 (0.96-1.87)	1.34 (0.94-1.90)
CC		24/19		20.57 ± 10.56		1.26 (0.66-2.41)	1.28 (0.65-2.52)
*rs2282679*	GC		0.890		**0.002**		
TT		167/127		20.34 ± 9.23		1.00	1.00
GT		164/116		18.05 ± 7.84		0.93 (0.67-1.30)	0.89 (0.63-1.25)
GG		32/25		17.16 ± 8.04		1.03 (0.58-1.82)	1.06 (0.59-1.91)
*rs3829251*	DHCR7		0.834		0.399		
GG		157/106		18.75 ± 8.82		1.00	1.00
GA		179/132		19.25 ± 8.39		1.09 (0.78-1.53)	1.06 (0.75-1.51)
AA		23/18		20.77 ± 11.10		1.16 (0.60-2.25)	1.23 (0.61-2.46)
*rs6013897*	CYP24A1		0.306		0.462		
TT		268/193		19.36 ± 9.06		1.00	1.00
AT		105/69		18.42 ± 7.68		0.91 (0.64-1.30)	0.91 (0.63-1.32)
AA		6/9		20.22 ± 9.91		2.08 (0.73-5.95)	2.25 (0.76-6.65)
*rs6599638*	C10orf88		0.409		**0.020**		
GG		87/65		19.77 ± 8.55		1.00	1.00
GA		179/147		19.37 ± 8.93		1.10 (0.75-1.62)	1.07 (0.71-1.60)
AA		88/55		17.16 ± 7.89		0.84 (0.53-1.33)	0.79 (0.49-1.28)

Adjusted OR is adjusted for age, prepregnancy BMI, family history of diabetes, parity, smoking, drinking, income, education, and conception season. Abbreviations: BMI: body mass index; OR: odds ratio; CI: confidence interval. ^∗^ *P* for the chi-square test. ^†^ *P* for Student's *t*-test/ANOVA

## Data Availability

The data used to support the findings of this study are available from the corresponding author upon request.

## Supplementary Materials

Supplementary Materials Supplementary Table 1: vitamin D concentration in the first trimester according to different maternal characteristics. Supplementary Table 2: linear regression for factors influences concentration of vitamin D in the first trimester. Supplementary Table 3: stratified analysis of association of vitamin D and the risk of GDM according to different genotypes.

## References

[1] Zhu Y., Zhang C. (2016). Prevalence of gestational diabetes and risk of progression to type 2 diabetes: a global perspective. *Current Diabetes Reports*.

[2] American Diabetes Association (2014). Standards of medical care in diabetes—2014. *Diabetes Care*.

[3] Bellamy L., Casas J. P., Hingorani A. D., Williams D. (2009). Type 2 diabetes mellitus after gestational diabetes: a systematic review and meta-analysis. *Lancet*.

[4] Malcolm J. C., Lawson M. L., Gaboury I., Lough G., Keely E. (2006). Glucose tolerance of offspring of mother with gestational diabetes mellitus in a low-risk population. *Diabetic Medicine*.

[5] Chiefari E., Arcidiacono B., Foti D., Brunetti A. (2017). Gestational diabetes mellitus: an updated overview. *Journal of Endocrinological Investigation*.

[6] Bendik I., Friedel A., Roos F. F., Weber P., Eggersdorfer M. (2014). Vitamin D: a critical and essential micronutrient for human health. *Frontiers in Physiology*.

[7] Holmes V. A., Barnes M. S., Alexander H. D., McFaul P., Wallace J. M. W. (2009). Vitamin D deficiency and insufficiency in pregnant women: a longitudinal study. *The British Journal of Nutrition*.

[8] Baker A. M., Haeri S., Camargo C. A., Stuebe A. M., Boggess K. A. (2012). First-trimester maternal vitamin D status and risk for gestational diabetes (GDM) a nested case-control study. *Diabetes/Metabolism Research and Reviews*.

[9] Lacroix M., Battista M. C., Doyon M. (2014). Lower vitamin D levels at first trimester are associated with higher risk of developing gestational diabetes mellitus. *Acta Diabetologica*.

[10] Zhang Y., Gong Y., Xue H., Xiong J., Cheng G. (2018). Vitamin D and gestational diabetes mellitus: a systematic Review based on data free of Hawthorne effect. *BJOG: An International Journal of Obstetrics & Gynaecology*.

[11] Yap C., Cheung N. W., Gunton J. E. (2014). Vitamin D supplementation and the effects on glucose metabolism during pregnancy: a randomized controlled trial. *Diabetes Care*.

[12] Torloni M. R., Betrán A. P., Horta B. L. (2009). Prepregnancy BMI and the risk of gestational diabetes: a systematic review of the literature with meta-analysis. *Obesity Reviews*.

[13] Bolland M. J., Grey A. B., Ames R. W. (2007). The effects of seasonal variation of 25-hydroxyvitamin D and fat mass on a diagnosis of vitamin D sufficiency. *American Journal of Clinical Nutrition*.

[14] O’Brien E. C., O’Sullivan E. J., Kilbane M. T., Geraghty A. A., McKenna M. J., McAuliffe F. M. (2017). Season and vitamin D status are independently associated with glucose homeostasis in pregnancy. *Nutrition & Metabolism*.

[15] PARK S., YOON H. K., RYU H. M. (2014). Maternal vitamin D deficiency in early pregnancy is not associated with gestational diabetes mellitus development or pregnancy outcomes in Korean pregnant women in a prospective study. *Journal of Nutritional Science and Vitaminology*.

[16] Shi A., Wen J., Liu G. (2016). Genetic variants in vitamin D signaling pathways and risk of gestational diabetes mellitus. *Oncotarget*.

[17] Wang Y., Wang O., Li W. (2015). Variants in vitamin D binding protein gene are associated with gestational diabetes mellitus. *Medicine*.

[18] Zhu B., Liang C., Xia X. (2018). Iron-related factors in early pregnancy and subsequent risk of gestational diabetes mellitus: the Ma’anshan birth cohort (MABC) study. *Biological Trace Element Research*.

[19] American Diabetes Association (2013). Standards of medical Care in Diabetes—2013. *Diabetes Care*.

[20] Zhou B. F. (2002). Predictive values of body mass index and waist circumference for risk factors of certain related diseases in Chinese adults--study on optimal cut-off points of body mass index and waist circumference in Chinese adults. *Biomedical and environmental sciences: BES*.

[21] Josefson J. L., Reisetter A., Scholtens D. M. (2016). Maternal BMI associations with maternal and cord blood vitamin D levels in a north American subset of hyperglycemia and adverse pregnancy outcome (HAPO) study participants. *PLoS One*.

[22] Zhou J., Su L., Liu M. (2014). Associations between 25-hydroxyvitamin D levels and pregnancy outcomes: a prospective observational study in southern China. *European Journal of Clinical Nutrition*.

[23] Pleskačová A., Bartáková V., Pácal L. (2015). Vitamin D status in women with gestational diabetes mellitus during pregnancy and postpartum. *BioMed Research International*.

[24] Casey C., McGinty A., Holmes V. A. (2018). Maternal vitamin D and markers of glycaemia during pregnancy in the Belfast Centre of the Hyperglycaemia and adverse pregnancy outcome study. *Diabetic Medicine*.

[25] Kramer C. K., Swaminathan B., Hanley A. J. (2014). Vitamin D and parathyroid hormone status in pregnancy: effect on insulin sensitivity, *β*-cell function, and gestational diabetes mellitus. *The Journal of Clinical Endocrinology & Metabolism*.

[26] Makgoba M., Nelson S. M., Savvidou M., Messow C. M., Nicolaides K., Sattar N. (2011). First-trimester circulating 25-hydroxyvitamin D levels and development of gestational diabetes mellitus. *Diabetes Care*.

[27] Savvidou M. D., Akolekar R., Samaha R. B., Masconi A. P., Nicolaides K. H. (2011). Maternal serum 25-hydroxyvitamin D levels at 11(+0) -13(+6) weeks in pregnant women with diabetes mellitus and in those with macrosomic neonates. *BJOG: An International Journal of Obstetrics & Gynaecology*.

[28] Aloia J. F. (2011). The 2011 report on dietary reference intake for vitamin D: where do we go from here?. *The Journal of Clinical Endocrinology & Metabolism*.

[29] Chiefari E., Pastore I., Puccio L. (2017). Impact of seasonality on gestational diabetes mellitus. *Endocrine, Metabolic & Immune Disorders - Drug Targets*.

[30] Verburg P. E., Tucker G., Scheil W., Erwich J. J. H. M., Dekker G. A., Roberts C. T. (2016). Seasonality of gestational diabetes mellitus: a south Australian population study. *BMJ Open Diabetes Research & Care*.

[31] Moses R. G., Wong V. C. K., Lambert K., Morris G. J., San Gil F. (2016). Seasonal changes in the prevalence of gestational diabetes mellitus. *Diabetes Care*.

[32] Katsarou A., Claesson R., Ignell C., Shaat N., Berntorp K. (2016). Seasonal Pattern in the Diagnosis of Gestational Diabetes Mellitus in Southern Sweden. *Journal of Diabetes Research*.

[33] Tucker P., Gilliland J. (2007). The effect of season and weather on physical activity: a systematic review. *Public Health*.

[34] Kan H., London S. J., Chen G. (2008). Season, sex, age, and education as modifiers of the effects of outdoor air pollution on daily mortality in Shanghai, China: the public health and air pollution in Asia (PAPA) study. *Environmental Health Perspectives*.

[35] Pevet P. (2003). Melatonin: from seasonal to circadian signal. *Journal of Neuroendocrinology*.

[36] McMullan C. J., Schernhammer E. S., Rimm E. B., Hu F. B., Forman J. P. (2013). Melatonin secretion and the incidence of type 2 diabetes. *JAMA*.

[37] Cooper J. D., Smyth D. J., Walker N. M. (2011). Inherited variation in vitamin D genes is associated with predisposition to autoimmune disease type 1 diabetes. *Diabetes*.

[38] Wang Y., Zhu J., DeLuca H. F. (2012). Where is the vitamin D receptor?. *Archives of Biochemistry and Biophysics*.

[39] Uitterlinden A. G., Fang Y., van Meurs J. B. J., Pols H. A. P., van Leeuwen J. P. T. M. (2004). Genetics and biology of vitamin D receptor polymorphisms. *Gene*.

[40] Al-Daghri N. M., Al-Attas O. S., Alkharfy K. M. (2014). Association of VDR-gene variants with factors related to the metabolic syndrome, type 2 diabetes and vitamin D deficiency. *Gene*.

[41] Ferrarezi D. A. F., Bellili-Muñoz N., Dubois-Laforgue D. (2013). Allelic variations of the vitamin D receptor (VDR) gene are associated with increased risk of coronary artery disease in type 2 diabetics: the DIABHYCAR prospective study. *Diabetes & Metabolism*.

[42] Grundberg E., Brandstrom H., Ribom E. L., Ljunggren O., Mallmin H., Kindmark A. (2004). Genetic variation in the human vitamin D receptor is associated with muscle strength, fat mass and body weight in Swedish women. *European Journal of Endocrinology*.

[43] Han F. F., Lv Y. L., Gong L. L., Liu H., Wan Z. R., Liu L. H. (2017). VDR gene variation and insulin resistance related diseases. *Lipids in Health and Disease*.

[44] Miettinen M. E., Smart M. C., Kinnunen L. (2015). Maternal VDR variants rather than 25-hydroxyvitamin D concentration during early pregnancy are associated with type 1 diabetes in the offspring. *Diabetologia*.

