# Supplementary material for: VDR Variants rather than Early Pregnancy Vitamin D Concentrations Are Associated with the Risk of Gestational Diabetes: The Ma'anshan Birth Cohort (MABC) Study
Source: J Diabetes Res. 2019 Jun 24;2019:8313901. doi: 10.1155/2019/8313901 (PMC6613005; doi:10.1155/2019/8313901)
Supplement: Supplementary Materials — Supplementary Table 1: vitamin D concentration in the first trimester according to different maternal characteristics. Supplementary Table 2: linear regression for factors influences concentration of vitamin D in the first trimester. Supplementary Table 3: stratified analysis of association of vitamin D and the risk of GDM according to different genotypes. [file 8313901.f1.pdf]

Supplementary Table 1. Vitamin D concentration in the first trimester according different maternal characteristics.

Variables	Vitamin D (Mean ± SD)	*P*
**Age**		**0.029**
<25	18.3 ± 8.3	
25~30	18.0 ± 8.3	
30~35	18.7 ± 8.4	
≥35	20.1 ± 9.8	
**BMI**		**0.014**
<18.5	18.0 ± 8.8	
18.5~24	18.1 ± 8.2	
24~28	18.7 ± 8.8	
≥28	21.2 ± 8.3	
**Parity**		**<0.001**
Nulliparous	18.0 ± 8.3	
Multiparous	19.9 ± 9.2	
**Education**		**0.015**
Primary school or below	19.8 ± 8.8	
Middle school	19.1 ± 8.7	
High school	18.5 ± 8.6	
Junior college	17.9 ± 8.1	
Undergraduate or above	17.8 ± 8.2	
**Monthly income (Chinese Yuan)**		0.666
<1000	18.6 ± 9.2	
1000~2500	18.2 ± 8.4	
2500~4000	18.1 ± 8.3	
>4000	18.5 ± 8.5	
**Smoking in early pregnancy**		**0.044**
No	18.3 ± 8.4	
Yes	16.8 ± 7.7	
**Drinking**		0.940
Never	18.2 ± 8.4	
Occasionally	18.2 ± 8.4	
Regularly	17.0 ± 5.3	
**Seasons of blood draw**		**<0.001**
Spring	16.8 ± 7.3	
Summer	21.1 ± 8.6	
Autumn	17.0 ± 7.9	
Winter	15.6 ± 8.0	
**Multi-vitamin intake before pregnancy**		**<0.001**
Never	18.0 ± 8.3	

1~2 times/week	18.5 ± 8.1	
3~6 times/week	20.3 ± 10.5	
Everyday	21.7 ± 9.8	
**Multi-vitamin intake in early pregnancy**		**<0.001**
Never	17.2 ± 7.8	
1~2 times/week	20.2 ± 7.6	
3~6 times /week	20.6 ± 9.2	
Everyday	22.0 ± 9.6	

Supplementary Table 2. Linear regression for factors influence concentration of vitamin D in the first trimester.

**Variable**	**Coefficient**	**Lower**	**Upper**	***P***
**Gestational weeks**	0.15	0.02	0.29	**0.024**
**Parity**	1.30	0.30	2.30	**0.011**
**Age**	0.01	-0.08	0.10	0.813
**BMI**	0.16	0.06	0.26	**0.003**
**Education**	-0.58	-0.86	-0.29	**<0.001**
**Income**	0.26	-0.11	0.64	0.170
**Smoking**	-1.76	-3.25	-0.27	**0.020**
**Drinking**	-0.15	-1.20	0.90	0.779
**Multi-vitamin intake before pregnancy**	0.34	-0.10	0.77	0.128
**Multi-vitamin intake in early pregnancy**	1.63	1.37	1.90	**<0.001**
**Season**	-1.00	-1.27	-0.73	**<0.001**

Supplementary Table 3. Stratified analysis of association of vitamin D and the risk of GDM according to different genotypes.

**Genotypes**	**Vitamin D**	**Adjusted OR (95%CI)**	***P* for interactions**
**rs1544410**			0.313
CC			
	Normal	1.00	
	Insufficiency	0.93 (0.55-1.58)	
CT			
	Normal	1.00	
	Insufficiency	0.27 (0.02-4.87)	
**rs731236**			0.693
AA			
	Normal	1.00	
	Insufficiency	0.89 (0.52-1.52)	
GA			
	Normal	1.00	
	Insufficiency	1.53 (0.05-45.94)	

**rs7041**			0.190
AA			
	Normal	1.00	
	Insufficiency	1.36 (0.58-3.23)	
CA/CC			
	Normal	1.00	
	Insufficiency	0.59 (0.29-1.20)	
**rs2282679**			0.481
TT	Normal	1.00	
	Insufficiency	0.67 (0.31-1.41)	
GT/GG	Normal	1.00	
	Insufficiency	1.22 (0.56-2.68)	
**rs3829251**			0.615
GG			
	Normal	1.00	
	Insufficiency	1.05 (0.47-2.34)	
GA/AA			
	Normal	1.00	
	Insufficiency	0.81 (0.39-1.65)	
**rs6013897**			0.688
TT			
	Normal	1.00	
	Insufficiency	0.91 (0.50-1.64)	
AT/AA			
	Normal	1.00	
	Insufficiency	0.79 (0.26-2.41)	
**rs6599638**			0.641
GG			
	Normal	1.00	
	Insufficiency	1.05 (0.38-2.90)	
GA/AA			
	Normal	1.00	
	Insufficiency	0.83 (0.43-1.59)	

Adjusted OR is adjusted for age, pre-pregnancy BMI, family history of diabetes, parity, smoking, drinking, gestational week of blood withdrawn, income, education and conception season.
